# Snoring Is Associated With Increased Risk of Stroke: A Cumulative Meta-Analysis

**DOI:** 10.3389/fneur.2021.574649

**Published:** 2021-04-01

**Authors:** Jing Bai, Bing He, Nan Wang, Yifei Chen, Junxiang Liu, Haoran Wang, Dongliang Liu

**Affiliations:** ^1^Cardiovascular Institute of Luohe, Luohe Central Hospital, Luohe Medical College, Luohe, China; ^2^Department of Clinical Medicine, Henan University of Science and Technology, Luohe, China

**Keywords:** snoring, stroke, meta-analysis, risk factors, public health

## Abstract

**Background:** Several studies have suggested that snoring is associated with an increased risk of stroke; however, the results are inconsistent. We aim to conduct a systematic review and meta-analysis of observational studies assessing the association between snoring and the risk of stroke in adults.

**Methods:** We searched PubMed for relevant studies. A random-effect model was adopted to summary relative risks (RRs), and forest plots from a cumulative meta-analysis method were used for a better presentation of how the pooled RRs changed as updated evidence accumulated.

**Results:** The literature search yielded 16 articles that met our inclusion criteria, and a total of 3,598 stroke patients and 145,901 participants were finally included in our analysis. A consistent trend toward association was found after the initial discovery, and the summary analysis indicated that snoring is associated with a 46% (RR, 1.46; 95%CI, 1.29–1.63; *p* < 0.001) increased risk of stroke.

**Conclusions:** Snoring is associated with a significantly increased risk for stroke, up to 46%. The importance of the current study lies in that we provide an imputes to take a more active approach against the increased risk of stroke in snorers.

## Background

Stroke is one of the leading causes of morbidity and mortality worldwide. Snoring, on the other hand, is one of the most common sleep disorders in the general population, with an estimated prevalence of about 30% in adults (1–3). Snoring is caused by soft tissue vibration of the upper airway. The presence of snoring indicates the airway is encroached by soft tissue, and the obstruction ranged from simple primary snoring through to mild, moderate, and severe sleep apnea. While the apnea associated increased risk of stroke has long been the subject of study and is relatively well-quantified (4, 5) the potential risk of snoring itself has been less aggressively studied, and the results reminded controversially. While several studies found that snoring is linked to a higher risk of stroke, negative results were also reported in other studies (6). A previous meta-analysis that incorporated six prospective studies found that a modest association between snoring and risk of stroke. However, many retrospective studies on this topic were not included in the analysis. And several large-scale and high-quality studies have been conducted [e.g., (6, 7)] after the publication of the above-mentioned meta-analysis. Adding these studies into an updated meta-analysis would augment the patient sample size, increase the precision of the effect size estimates, facilitate the power of moderator analyses, and possibly change the direction of the association. Therefore, we conducted an updated meta-analysis to clarify the relationship between snoring and stroke. We also performed subgroup analysis by different population features and study characteristics.

## Methods

The focused question of this quantitative review was developed with the PICO tool, and the study was performed following Preferred reporting items for systematic review and meta-analysis (PRISMA) guidelines for the development of protocols and reporting all the necessary items (8, 9).

### Search Strategy and Selection Criteria

All studies reporting the relationship of snoring with stroke were identified by a thorough search of the databases including the Cochrane Library, Medline, and Embase. The search terms were: (“snoring” OR “snorers”) AND (“stroke” OR “cerebrovascular accident” OR “brain ischemia” OR “brain infarction” OR “cerebral hemorrhage” OR “intracerebral hemorrhage”). Besides, we checked the references of all the retrieved articles to identify further relevant articles.

The eligibility of studies was judged according to the elements of PICO. Participants: Adults with a clear description of the status of snoring, either by self-report or questionnaire, or reported by roommate; Intervention: no intervention; Comparison: snorers vs. non-snorers; Outcome: all types of stroke, including ischemic stroke and hemorrhage stroke, with a clear description of the ascertainment of stroke diagnosis (referring to medical record or by self-report history). Additionally, studies should include reported estimates [risk ratio (RR), hazards ratio (HR), or odds ratio (OR)] and corresponding 95% CIs describing the association between snoring and risk of stroke. We excluded studies including adolescents; systematic reviews, meta-analysis, and studies carried out in non-humans were also excluded.

### Data Extraction and Quality Assessment

All data were extracted independently by two reviewers (J.B. and B.H.) with a predesigned data abstraction form. The following information was extracted or calculated from the included articles: study design, authors, publication year, country, numbers of total participants and stroke patients, gender, and adjustments. The results were compared, and differences were resolved by discussion and consensus with a third reviewer (H.W.).

### Quality Assessment

We assessed the quality of the included studies by employing the Newcastle Ottawa Scale (NOS) (10) which judges the methodological quality. A full score is 11, and a score of 9 or more was regarded as “high quality”; otherwise, the study was regarded as “low quality.” Any disagreements on the NOS score of the studies were resolved through a comprehensive reassessment by the other authors.

### Statistical Analysis

We extracted the risk estimates and their 95% confidence intervals for the association results. If risk estimates from several statistical models were presented in one study, we will choose the model that included most confounders. To calculate the pooled estimate, the ORs in retrospective studies were converted to RRs with the formula RR = OR/[[1–pRef] + [pRef^*^OR]], in which pRef refers to the prevalence of snoring in the control group (11). The between-study heterogeneity was assessed by the I^2^ static, and heterogeneity was considered to be significant if the I^2^ was over 50%. The summary RR was calculated through the random-effects model, which takes into account the presence of heterogeneity in their calculations (12). We applied the cumulative meta-analysis method to present the result because this method takes advantage of reducing the risk of false-positive when performing repetitive estimates (13). Besides, the stability, and reliability of the pooled estimate were assessed by sensitivity analyses, in which we omitted one estimate at a time sequentially and recalculated the pooled results. The stability and reliability were confirmed if no single study altered the significance of the pooled estimate. Furthermore, publication bias was assessed through Begger's funnel plot and Egger's linear regression test, and a *P* < 0.05 was considered significant (14). The statistical analysis was performed with STATA software (Version 13.3; Stata Corporation, College Station, TX, USA). All *P*-values were two-tailed.

## Results

### Characteristics of Included Studies

**Figure 1:**
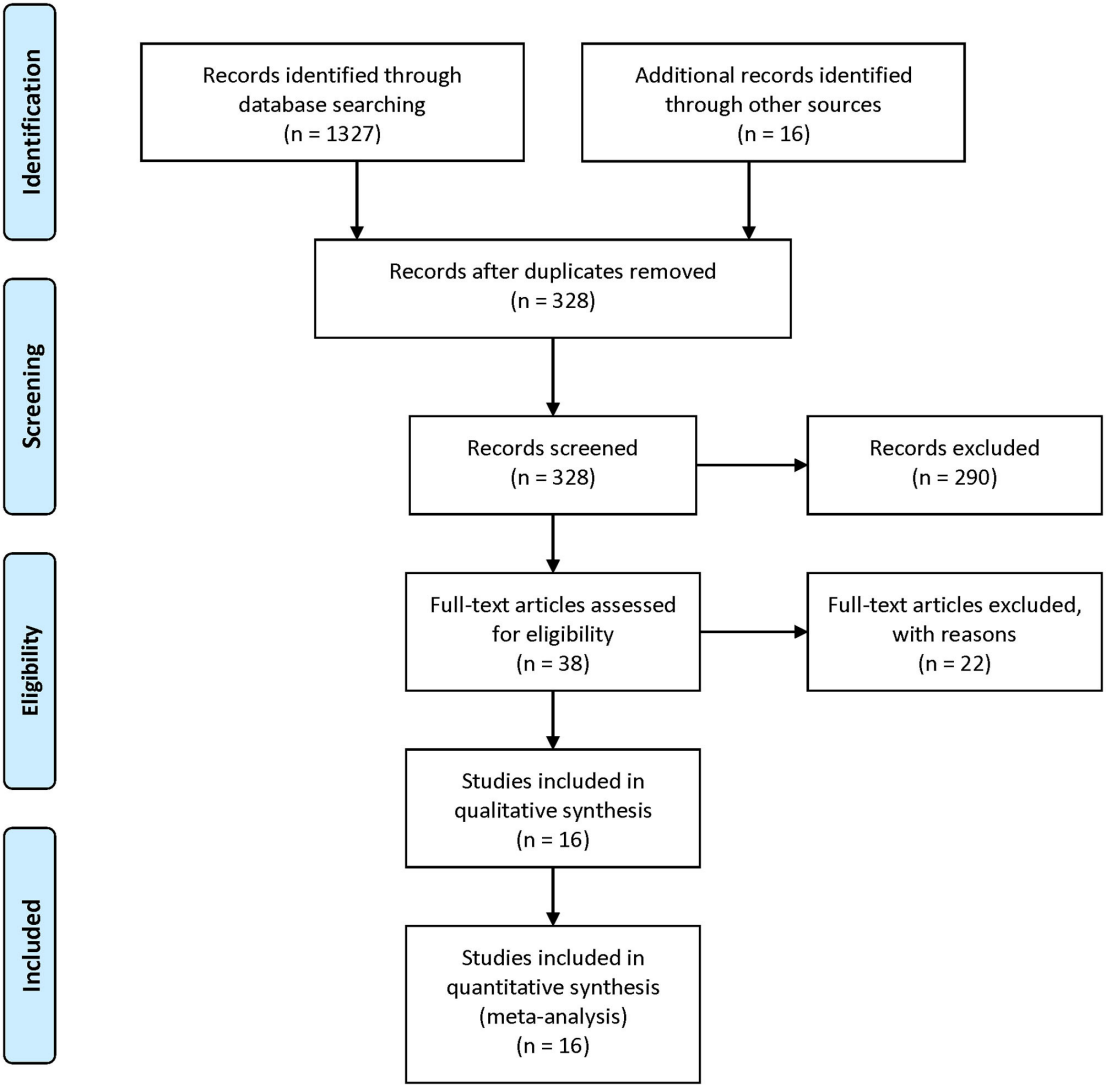
PRISMA Flow-diagram depicting identification and selection process for the present meta-analysis.

Our literature screening process is shown in Figure 1. Based on our selection strategy, a total of 1,134 articles were retrieved from the databases and reference lists at the initial screening process. After we removed the articles which did not meet the inclusion criteria, 16 studies were left, with 3,598 stroke patients and 145,901 participants finally included in our analysis (6, 7, 15–28). Of these included studies, four were conducted in U.S. (18, 21, 24, 28) three in U.K. (15, 17, 26) three in Finland (20, 22, 23) and one study conducted in Italy (25), Denmark (19), France (27), Hungary (16), Australia (6), and China (7), respectively. Nine studies included men and women (6, 7, 15, 16, 19, 24, 25, 27, 28) a single study reported the risk estimates for men and women separately (7) five studies included only men (17, 20, 22, 23, 26) and two studies included only women (18, 21). In 11 studies stroke refers to ischemic stroke and hemorrhagic stroke together (6, 7, 15, 16, 18–20, 24–26, 28). In five studies it refers to ischemic stroke (17, 21–23, 27); two studies reported the risk estimates for ischemic stroke and hemorrhagic stroke separately (7, 18). Table 1 summarizes the basic information of each study, including the authors, year of publication, gender, country, sample size, adjustments, and so on. The NOS scores of each included study were listed in Supplementary Table 2. Overall, the level was adequate.

**Table 1:** Characteristics of included studies.

**References**	**Country**	**Cohort name**	**Total participants**	**Total Stroke patients**	**Adjustment**
Koskenvuo et al. (20)	Finland	Finnish twin cohort study	4,388	42	Age, hypertension, BMI
Partinen and Palomaki (23)	Finland	NA	150	50	Age, BMI
Schmidt-Nowara et al. (24)	U.S.	NA	1,206	45	Age, gender, obesity, and smoking
Palomäki (22)	Finland	NA	354	177	Diabetes mellitus, obesity, smoking, frequent drinking
Spriggs et al. (26)	U.K.	NA	671	326	Obesity, smoking, drinking alcohol, history of cerebrovascular disease, ischemic heart disease, hypertension, atrial fibrillation, and diabetes
Smirne et al. (25)	Italy	NA	300	164	Age, sex, smoking, alcohol, BMI, diabetes, dyslipidemia, hypertension
Jennum et al. (19)	Denmark	Copenhagen Male Study	2,937	60	Age, tobacco use, alcohol consumption, and BMI.
Neau et al. (27)	France	NA	266	133	Arterial hypertension, coronary heart disease, diabetes mellitus, arteriopathy of the lower limbs, smoking, alcohol consumption, body mass index
Hu et al. (18)	U.S.	Nurses' Health Study	71,779	398	Age; time period; body mass index; cigarette smoking; menopausal status; parental history of myocardial infarction before 60 years of age; alcohol consumption; multivitamin and vitamin E supplement use; physical activity; average number hours of sleeping; usual sleep positions; history of diabetes; history of hypercholesterolemia.
Davies et al. (15)	U.K.	NA	362	181	Smoking, alcohol, hypertension, Epworth score
Elwood et al. (17)	U.K.	Caerphilly cohort	1,986	106	Age, social class, smoking, alcohol consumption, BMI, and neck circumference.
Dunai et al. (16)	Hungary	Hungaro study 2002	12,643	487	Age, sex, body mass index, diabetes, education, smoking status, and alcohol consumption.
Yeboah et al. (28)	U.S.	MESA	5,338	79	Age, gender, race/ethnicity, BMI, cigarette smoking, diabetes mellitus, total cholesterol, HDL, triglycerides, systolic blood pressure, BP medication use, statin use, benzodiazepine use and current alcohol use.
Marshall et al. (6)	Australia	Busselton Health Study	397	24	Sleep apnea, age, sex, body mass index, smoking status, total cholesterol, high-density lipoprotein cholesterol, mean arterial pressure, diabetes, doctor-diagnosed angina.
Sands et al. (21)	U.S.	Women's Health Initiative	42,244	993	Age, race, education, income, smoking, physical activity, alcohol intake, depression, diabetes, high blood pressure, BMI, waist-to-hip ratio, hyperlipidemia
Wen et al. (7)	China	NA	880	333	Age, sex, sleep duration, daytime napping, snorting/gasping, education, smoking, alcohol, vegetables, fruits consumption status, history of diabetes, history of hypertension, BMI, physical activity, sleep quality, and psychosocial factors

### Meta-Analysis Results and Heterogeneity Analysis

**Figure 2:**
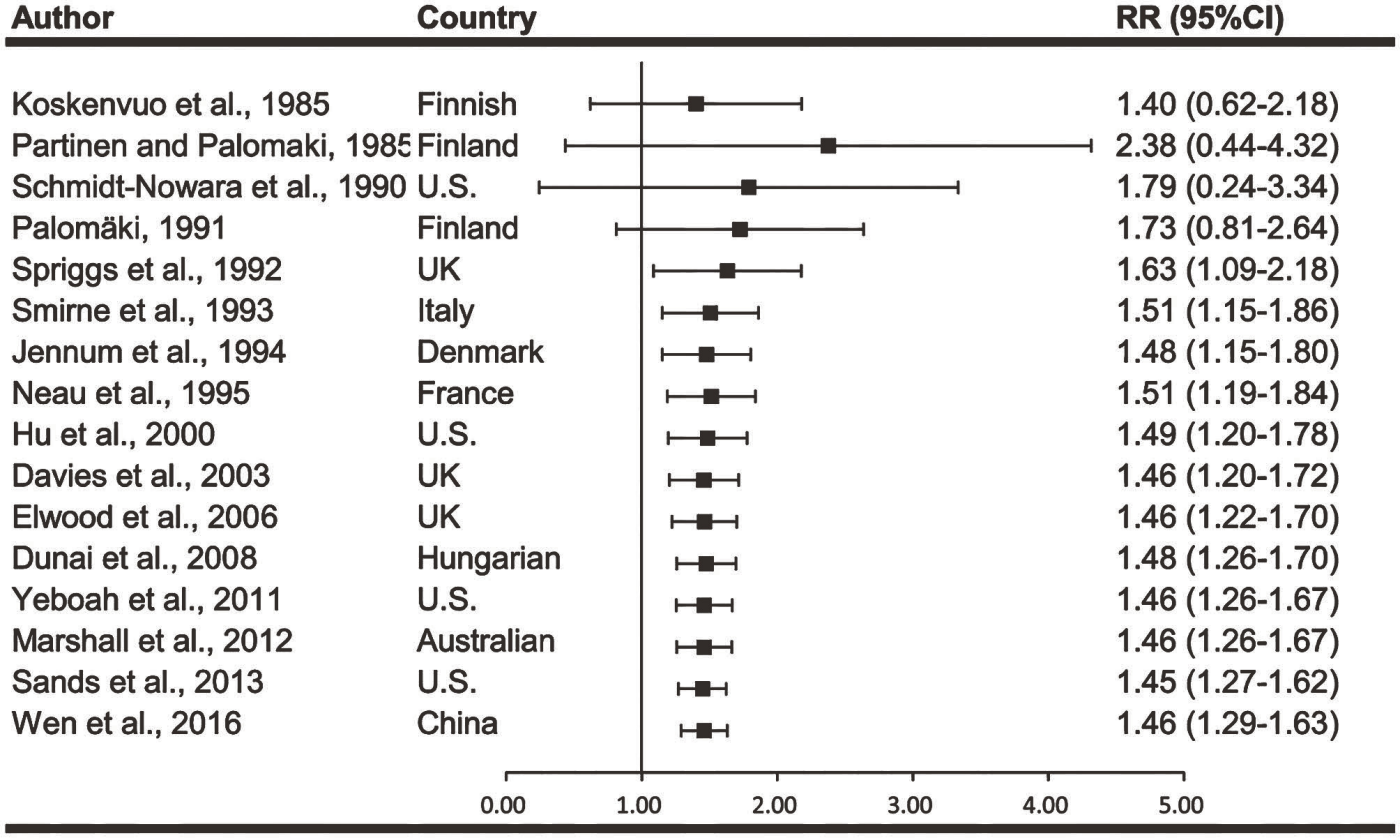
Cumulative meta-analysis describing the association between snoring and risk of stroke. The results indicated there is a consistent association after the initial discovery.

Meta-analyses are shown in Figure 2. When comparing effect sizes, we found significant heterogeneity in the analyses. Cumulative meta-analyses indicated there is a consistent trend toward association after the initial discovery. The pooled results showed that snoring associates with a 46% increased risk of stroke (RR, 1.46; 95%CI, 1.29–1.63; *p* < 0.001).

**Figure 3:**
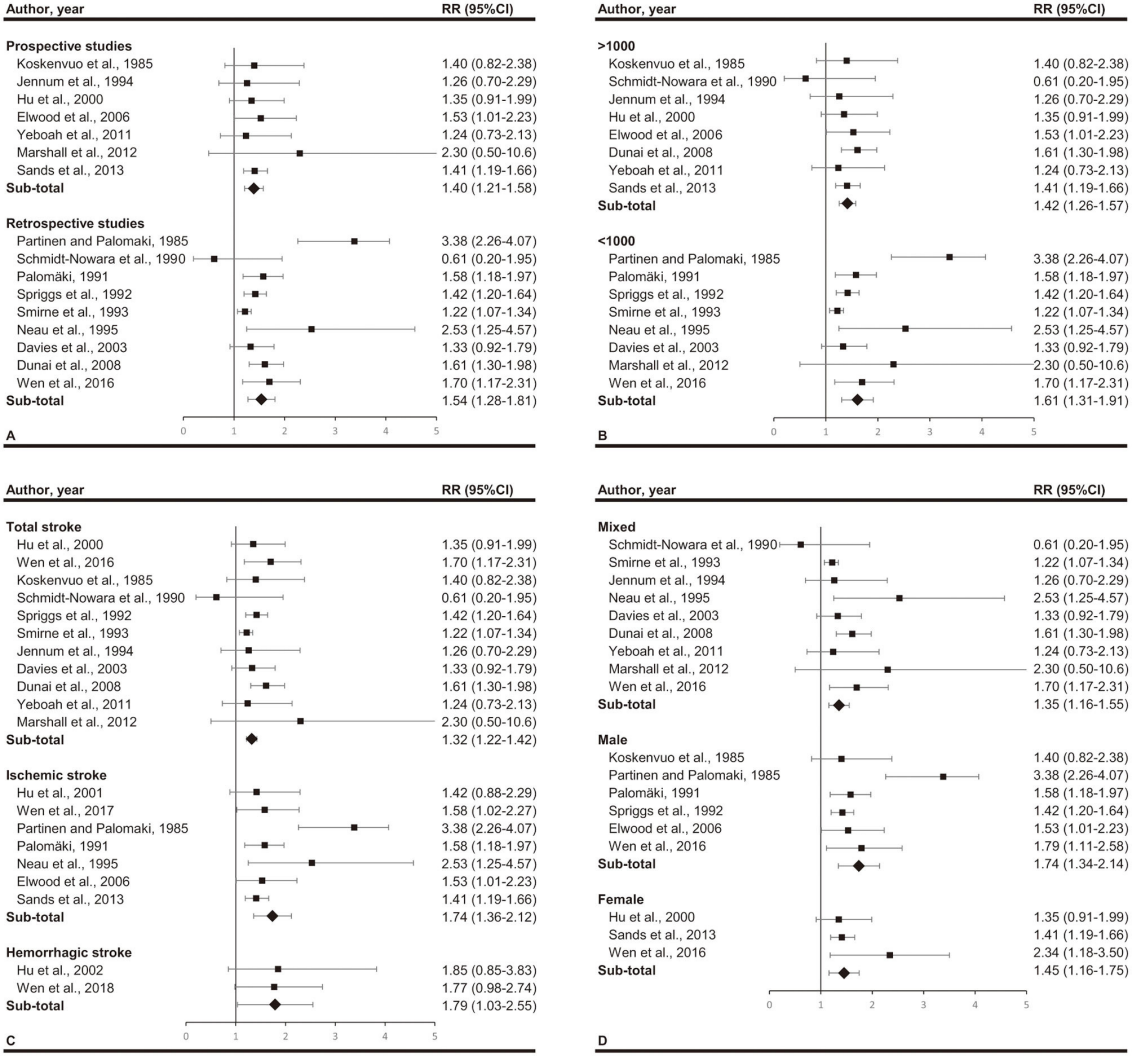
Subgroup analyses by **(A)** study design (prospective or retrospective), **(B)** total participants (over 1,000 or not), **(C)** stroke type (total stroke, ischemic stroke, and hemorrhagic stroke), and **(D)** gender (mixed, male, and female). Significant association between snoring and stroke exists in all the subgroups.

Subgroup analyses are illustrated in Figure 3. When subgroups were stratified by study design, we found significant association lies in both retrospective studies (RR, 1.54; 95% CI, 1.28–1.81; *p* < 0.001) and prospective studies (RR, 1.40; 95% CI, 1.21–1.58; *p* < 0.001). When subgroups were stratified by participants number, we found significant association lies in both larger studies (>1,000 participants) (RR, 1.42; 95% CI, 1.26–1.57; *p* < 0.001) and smaller studies (<1,000 participants) (RR, 1.61; 95% CI, 1.31–1.91; *p* < 0.001). The stratified analysis according to sex included 3 subgroups, male (RR, 1.74; 95% CI, 1.34–2.14; *p* < 0.001), female (RR, 1.45; 95% CI, 1.16–1.75; *p* < 0.001), and mix (RR, 1.35; 95% CI, 1.16–1.55; *p* < 0.001). When studies were stratified according to stroke type, we found a RR of 1.32 (95% CI, 1.22–1.42; *p* < 0.001) for total stroke, 1.74 (95%CI, 1.36–2.12; *p* < 0.001) for ischemic stroke and 1.79 (95%CI, 1.03–2.55; *p* < 0.001) for hemorrhagic stroke.

### Sensitivity Analysis and Publication Bias

**Figure 4:**
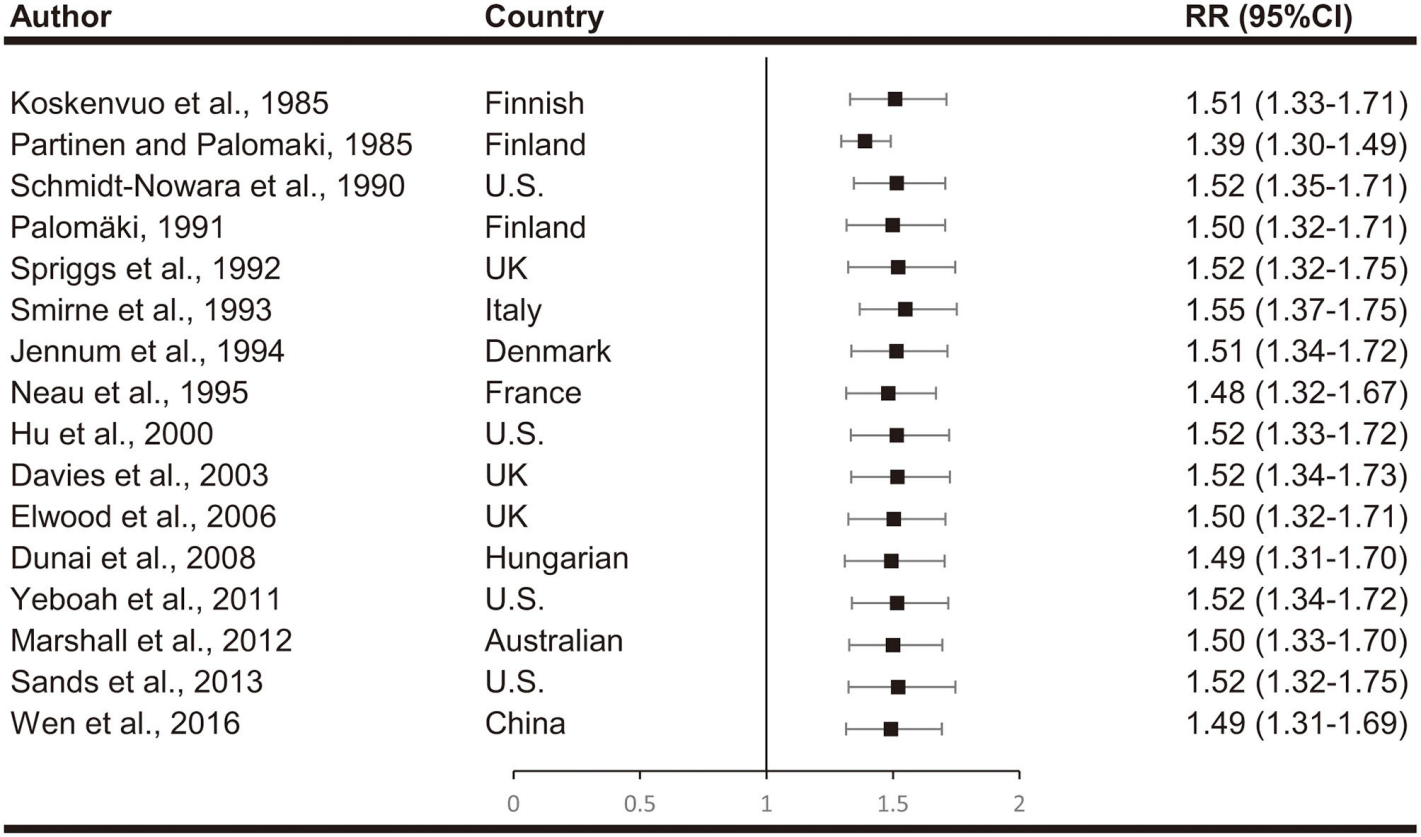
Sensitivity analyses by omitting one study at a time.

We estimated the stability of all the pooled ORs by eliminating all included studies one each time and recalculate the summary RR. The significance of the corresponding RRs was not altered, indicating that our results were stable (Figure 4).

**Figure 5:**
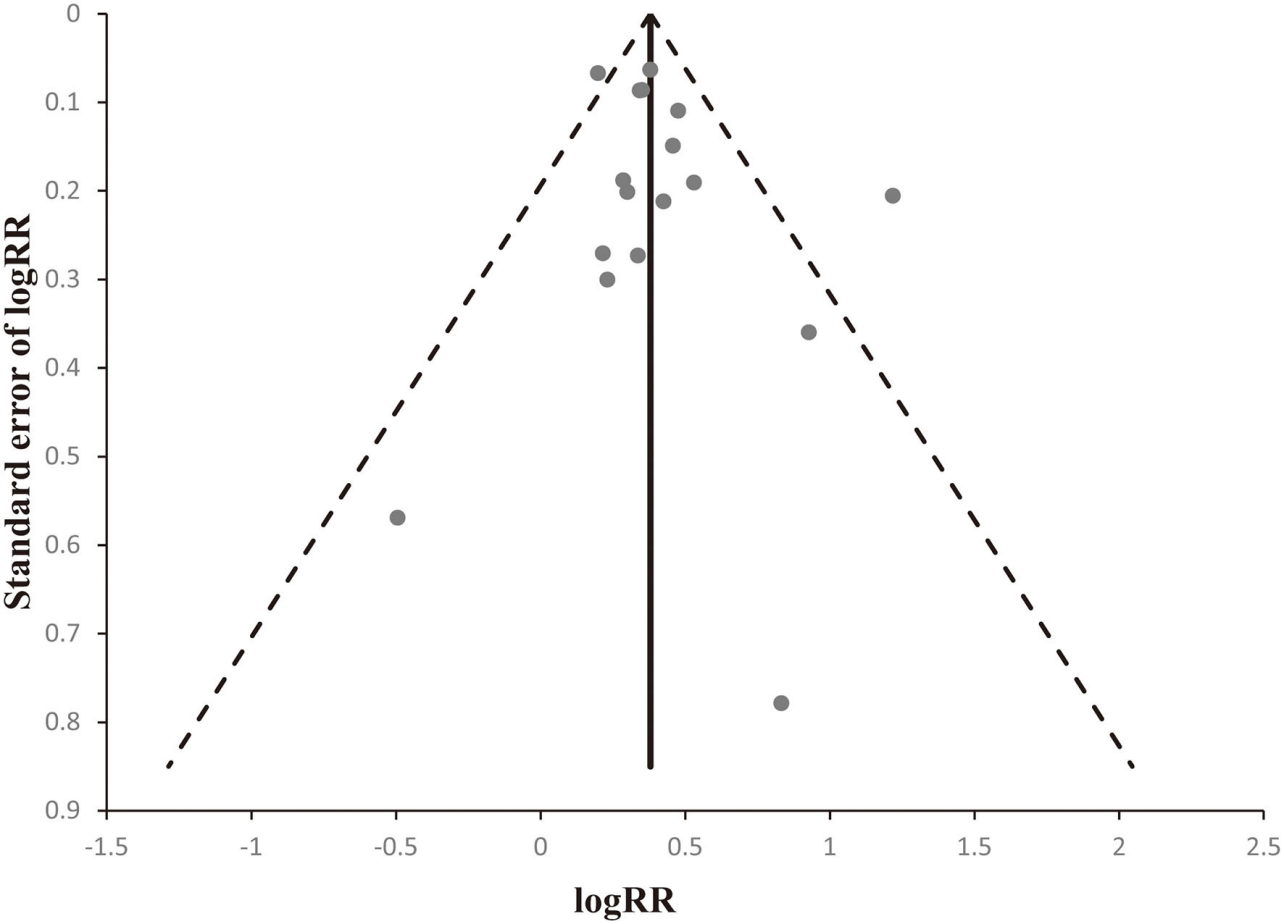
Begger's funnel plots to evaluate publication bias. The shape of the funnel plot was obviously symmetrical.

Egger's linear regression tests and funnel plots were applied to assess possible publication bias. The shape of the funnel plot was symmetrical (Figure 5). The *p*-value of Egger's test also suggested that there was no significant publication bias (t = 1.24, *p* = 0.236).

## Discussion

The association between snoring and stroke susceptibility has been studied for many years, however, the results are still controversial. As previous studies were limited by the small sample size, here we performed this meta-analysis to clarify the association by incorporating all available data. To our knowledge, this updated meta-analysis is the largest review, including nearly 150,000 individuals and 4,000 stroke patients. We found that snoring was associated with a 46% increase in the risk of stroke. A cumulative meta-analysis confirmed the reliability of the association, and further studies on this topic are less likely to affect the current conclusion. If a future study does report a contradictory result, adding it into the analysis will significantly increase the heterogeneity of the pooled analysis, which means the weight it receives will be reduced, and the conclusion is unlikely to be changed. Besides, stratified analyses according to study design, sample size, stroke type, and gender indicated that the association was consistent across subgroups.

Detailed mechanisms underlying the observed association between snoring and stroke are not completely clear, but it was suggested that snoring might contribute to the inducing and promoting of atherosclerosis. The most widely studied is the deleterious effect of sleep apnea and associated hypoxemia, which might introduce oxidative stress (29, 30), inflammation (31–33), insulin resistance (34–36), endothelial disfunction (37, 38), diabetes (39, 40), dyslipidemia (41, 42), and hypertension (43, 44) all of which may contribute to the development of atherosclerosis and predispose to stroke. Apart from that, the vibration from snoring may be another potential mechanism. Studies have found a high level of energy exists in the oscillating tissues during snoring which could transmit to proximal tissues including the carotid artery. And the vibration transmitted may trigger a cascade effect to the numerous cells of the arterial wall and change the structure and function of the vessel (45, 46). Taken together, these studies provided evidence that the snoring-related vibration might cause injury to the vessel, promote the formation of atherosclerotic plaque and provide force for the rupture of plaque (47).

Our findings implicated that the risk of developing stroke is not confined to the population of patients with established sleep apnea, but also extends to the population of snorers. Snoring is associated with sleep apnea but is much more common, with a prevalence in middle-aged adults of about 30%. While it has generally been regarded as a simple lifestyle disorder, the current analyses indicate that snoring is associated with a significantly increased risk of stroke. The importance of the current study lies in that we provide an imputes to take a more active approach against the increased risk of stroke in snorers.

When interpreting the results, several limitations should be noted. First, most of the included studies were carried out in Europe and the U.S., therefore it is not appropriate to generalize our findings to Asian and African populations. Second, although there are many participants included in the analysis, the individual participant data was not acquired. Besides, our meta-analysis was based on studies that varied in many ways including study design, population sample, adjustment for confounders, and different ascertainment methods for exposure and outcome, which may be considered another limitation. To minimize the effect of this limitation, we adopted appropriate meta-analytic techniques with random-effect models and cumulative analysis, which enabled us to account for these differences. The consistency of the evidence across time all subgroups support a real association between snoring and stroke risk. However, when interpreting the results of subgroup analysis, it should be noted that biological studies regarding the mechanism of the relationship between snoring and stroke are still lacking. Therefore, the detailed mechanism by which subgroup factors contribute to the increased risk of stroke due to snoring remains to be elucidated in future studies.

## Conclusions

In conclusion, this meta-analysis provides evidence that snoring may be a significant risk factor for stroke. Given the high prevalence of snoring and disease burden of stroke in the general population, taking a more active approach against the increased risk of stroke in snorers may be worthwhile.

## Data Availability Statement

The original contributions presented in the study are included in the article/Supplementary Material, further inquiries can be directed to the corresponding author/s.

## Author Contributions

HW and DL drafted the study protocol. JB, BH, and NW collected information. JB, NW, and YC performed data analysis. JB, BH, and JL drafted the paper. JB, HW, and DL had full access to all the data in the study and takes responsibility for the integrity of the information and the accuracy of the information. JB, HW, and DL are the guarantors of the paper. All authors have read and approved the manuscript and critically reviewed the paper.

## Conflict of Interest

The authors declare that the research was conducted in the absence of any commercial or financial relationships that could be construed as a potential conflict of interest.

## Supplementary Material

Supplementary Table 1 PRISMA-2009-Checklist.

Supplementary Table 2 Newcastle Ottawa Scale for quality assessment.

## Abbreviations

CI: confidence interval
NOS: Newcastle Ottawa Scale
OR: Odds ratio
PRISMA: Preferred reporting items for systematic review and meta-analysis
RR: Relative risk.
