# Supplementary material for: Snoring Is Associated With Increased Risk of Stroke: A Cumulative Meta-Analysis
Source: Front Neurol. 2021 Apr 1;12:574649. doi: 10.3389/fneur.2021.574649 (PMC8047148; doi:10.3389/fneur.2021.574649)
Supplement: Supplementary Table 2 — Newcastle Ottawa Scale for quality assessment. [file Table_2.DOCX]

**Table S2. Newcastle Ottawa Scale for quality assessment of retrospective studies.**

| Selection | Score | Partinen and Palomaki, 1985 | Schmidt-Nowara et al., 1990 | Palomäki, 1991 | Spriggs et al., 1992 | Smirne et al., 1993 | Neau et al., 1995 | Davies et al., 2003 | Dunai et al. 2008 | Wen et al., 2016 |
| --- | --- | --- | --- | --- | --- | --- | --- | --- | --- | --- |
| 1) Is the case definition adequate? |  |  |  |  |  |  |  |  |  |  |
| a) yes, based on medical record | 2 | 2 |  | 2 | 2 | 2 | 2 | 2 | 2 | 2 |
| b) yes, based on self-reports | 1 |  | 1 |  |  |  |  |  |  |  |
| c) no description | 0 |  |  |  |  |  |  |  |  |  |
| 2) Representativeness of the cases |  |  |  |  |  |  |  |  |  |  |
| a) consecutive or obviously representative series of cases | 1 | 1 | 1 | 1 | 1 | 1 | 1 | 1 | 1 | 1 |
| b) potential for selection biases or not stated | 0 |  |  |  |  |  |  |  |  |  |
| 3) Selection of Controls |  |  |  |  |  |  |  |  |  |  |
| a) community controls | 2 |  | 2 |  | 2 |  | 2 | 2 | 2 | 2 |
| b) hospital controls | 1 | 1 |  | 1 |  | 1 |  |  |  |  |
| c) no description | 0 |  |  |  |  |  |  |  |  |  |
| 4) Definition of Controls |  |  |  |  |  |  |  |  |  |  |
| a) no history of stroke | 1 | 1 | 1 | 1 | 1 | 1 | 1 | 1 | 1 | 1 |
| b) no description of source | 0 |  |  |  |  |  |  |  |  |  |
| Comparability |  |  |  |  |  |  |  |  |  |  |
| 1) Comparability of cases and controls on the basis of the design or analysis |  |  |  |  |  |  |  |  |  |  |
| a) study controls for age | 2 |  | 2 | 2 | 2 | 2 | 2 | 2 | 2 | 2 |
| b) study controls for any additional factor | 1 |  |  |  |  |  |  |  |  |  |
| c) no adjustment | 0 | 0 |  |  |  |  |  |  |  |  |
| Exposure |  |  |  |  |  |  |  |  |  |  |
| 1) Ascertainment of exposure |  |  |  |  |  |  |  |  |  |  |
| a) objectively assessed | 2 |  |  |  |  |  |  |  |  |  |
| b) self-reported | 1 | 1 | 1 | 1 | 1 | 1 | 1 | 1 | 1 | 1 |
| c) no description | 0 |  |  |  |  |  |  |  |  |  |
| 2) Same method of ascertainment for cases and controls |  |  |  |  |  |  |  |  |  |  |
| a) yes | 1 | 1 | 1 | 1 | 1 | 1 | 1 | 1 | 1 | 1 |
| b) no | 0 |  |  |  |  |  |  |  |  |  |
| Total score | 11 | 7 | 9 | 9 | 10 | 9 | 10 | 10 | 10 | 10 |
| Quality level* |  | Low | High | High | High | High | High | High | High | High |

**Table S2. Newcastle Ottawa Scale for quality assessment of prospective studies.**

| Selection | Score | Hu et al. 2000 | Koskenvuo et al. 1985 | Yeboah et al., 2011 | Marshall et al., 2012 | Zamarrón et al. 1999 | Jennum et al. 1994 | Sands et al. 2013 | Elwood et al. 2005 |
| --- | --- | --- | --- | --- | --- | --- | --- | --- | --- |
| 1) Representativeness of the exposed cohort |  |  |  |  |  |  |  |  |  |
| a) truly representative of the individuals exposed to snoring in the community | 2 | 2 | 2 | 2 | 2 | 2 | 2 | 2 | 2 |
| b) somewhat representative of the individuals exposed to snoring in the community | 1 |  |  |  |  |  |  |  |  |
| c) selected group of users eg nurses, volunteers | 0 |  |  |  |  |  |  |  |  |
| d) no description of the derivation of the cohort | 0 |  |  |  |  |  |  |  |  |
| 2) Selection of the non-exposed cohort |  |  |  |  |  |  |  |  |  |
| a) drawn from the same community as the exposed cohort | 2 | 2 | 2 | 2 | 2 | 2 | 2 | 2 | 2 |
| b) drawn from a different source | 1 |  |  |  |  |  |  |  |  |
| c) no description of the derivation of the non-exposed cohort | 0 |  |  |  |  |  |  |  |  |
| 3) Ascertainment of exposure |  |  |  |  |  |  |  |  |  |
| a) self-report | 1 | 1 | 1 | 1 | 1 | 1 | 1 | 1 | 1 |
| b) no description | 0 |  |  |  |  |  |  |  |  |
| 4) Demonstration that outcome of interest was not present at start of study |  |  |  |  |  |  |  |  |  |
| a) yes | 1 | 1 | 1 | 1 | 1 | 1 | 1 | 1 |  |
| b) no | 0 |  |  |  |  |  |  |  | 0 |
| Comparability |  |  |  |  |  |  |  |  |  |
| 1) Comparability of cohorts on the basis of the design or analysis |  |  |  |  |  |  |  |  |  |
| a) study controls for age and any additional factor | 2 | 2 | 2 | 2 | 2 | 2 | 2 | 2 | 2 |
| b) study controls for any confounding factor | 1 |  |  |  |  |  |  |  |  |
| c) no adjustment | 0 |  |  |  |  |  |  |  |  |
| Outcome |  |  |  |  |  |  |  |  |  |
| 1) Assessment of outcome |  |  |  |  |  |  |  |  |  |
| a) record linkage | 2 | 2 | 2 | 2 | 2 | 2 |  | 2 | 2 |
| b) self-report | 1 |  |  |  |  |  | 1 |  |  |
| c) no description | 0 |  |  |  |  |  |  |  |  |
| 2) Was follow-up long enough for outcomes to occur |  |  |  |  |  |  |  |  |  |
| a) yes (select an adequate follow up period for outcome of interest) | 1 | 1 | 1 | 1 | 1 | 1 | 1 | 1 | 1 |
| b) no | 0 |  |  |  |  |  |  |  |  |
| Total score | 11 | 11 | 11 | 11 | 11 | 11 | 10 | 11 | 10 |
| Quality level* |  | High | High | High | High | High | High | High | High |
